# The anti-apoptotic BAG3 protein is involved in BRAF inhibitor resistance in melanoma cells

**DOI:** 10.18632/oncotarget.18902

**Published:** 2017-06-30

**Authors:** Luana Guerriero, Giuseppe Palmieri, Margot De Marco, Antonio Cossu, Paolo Remondelli, Mario Capunzo, Maria Caterina Turco, Alessandra Rosati

**Affiliations:** ^1^ BIOUNIVERSA s.r.l., 84084 Baronissi, Italy; ^2^ Unit of Cancer Genetics, Institute of Biomolecular Chemistry (ICB), National Research Council (CNR), 07100 Sassari, Italy; ^3^ Unit of Pathology, Azienda Ospedaliero Universitaria (AOU), University di Sassari, 07100 Sassari, Italy; ^4^ Department of Medicine, Surgery and Dentistry “Scuola Medica Salernitana” University of Salerno, 84084 Baronissi, Italy

**Keywords:** BAG3, melanoma, BRAF, vemurafenib, resistance

## Abstract

BAG3 protein, a member of BAG family of co-chaperones, has a pro-survival role in several tumour types. BAG3 anti-apoptotic properties rely on its characteristic to bind several intracellular partners, thereby modulating crucial events such as apoptosis, differentiation, cell motility, and autophagy. In human melanomas, BAG3 positivity is correlated with the aggressiveness of the tumour cells and can sustain IKK-γ levels, allowing a sustained activation of NF-κB. Furthermore, BAG3 is able to modulate BRAFV600E levels and activity in thyroid carcinomas. BRAFV600E is the most frequent mutation detected in malignant melanomas and is targeted by Vemurafenib, a specific inhibitor found to be effective in the treatment of advanced melanoma. However, patients with BRAF-mutated melanoma may result insensitive *ab initio* or, mostly, develop acquired resistance to the treatment with this molecule.

Here we show that BAG3 down-modulation interferes with BRAF levels in melanoma cells and sensitizes them to Vemurafenib treatment. Furthermore, the down-modulation of BAG3 protein in an *in vitro* model of acquired resistance to Vemurafenib can induce sensitization to the BRAFV600E specific inhibition by interfering with BRAF pathway through reduction of ERK phosphorylation, but also on parallel survival pathways.

Future studies on BAG3 molecular interactions with key proteins responsible of acquired BRAF inhibitor resistance may represent a promising field for novel multi-drugs treatment design.

## INTRODUCTION

Melanoma incidence is steadily increasing worldwide and people affected by the metastatic form of this malignancy had a median survival time of 6–8 months [1]. In the last ten years, the discovery of BRAF mutations in melanoma created the first opportunity to develop oncogene-directed therapy, which had produced major clinical responses and significantly improved survivals [2, 3, 4]. Although the outstanding results on patients give hopes that melanoma can be cured, achievement of prolonged survivals is hampered by the appearance of resistance mechanisms that may quickly develop and lead to relapse in patients treated with BRAF inhibitors [5, 6]. Recently, clinical evidence of higher effectiveness of the combinatorial trials using BRAF inhibitors together with MEK and, to a less extent, PI3K inhibitors is providing further treatment options [7].

However, the issue of acquired resistance to BRAF inhibitor still remains a challenge [6]. Indeed, what is needed is a progress in understanding the multiple coexistent aberrations in resistant melanoma cells and addressing novel multi-target therapeutic modules to narrow the propensity for growth and spreading of resistant tumours.

BAG3 protein, a member of the family of heat shock protein (HSP) 70 co-chaperones that share the BAG domain, is expressed in a wide range of human tumours; in physiological conditions, its expression is conversely narrowed to few cell types (such as myocytes) [8, 9]. Recently, we reported that BAG3 levels in melanomas appeared to be specifically expressed in the cytoplasm of neoplastic cells while normal skin and benign nevi were negative [10]. More recently, we identified a subgroup of stage III melanoma patients, i.e. patients with 2–3 positive lymph nodes, whose clinical behaviour is influenced by the expression of the anti-apoptotic BAG3 protein in lymph node metastasis, suggesting that BAG3 staining on lymph node biopsies could therefore contribute to patient's prognosis and stratification for specific therapeutic approaches [11].

To BAG3 protein was assigned a role in sustaining the growth and in contributing to chemotherapy resistance in some tumour types [12, 13, 14, 15, 16]. We also demonstrated that, in melanoma cells, BAG3 is able to modulate the Hsp70- mediated delivery of the IKKγ subunit of IKK complex to proteasome, thereby sustaining NF-κB activation and inhibiting cell apoptosis. In a melanoma xenograft model, *bag3* silencing indeed resulted in a significant reduction of tumour growth with subsequent prolonged animal's survival [17]. Furthermore, it was reported that in thyroid cancer cells (harbouring BRAFV600E mutation) BAG3 can regulate cell growth both *in vitro* and *in vivo* and the underlying molecular mechanism appears to rely on BAG3 binding to BRAF, that protects BRAF from proteasome-dependent degradation [18].

Toward the elucidation of mechanisms by which resistance develops in treatment-resistant melanomas, we think that a contribution to this issue may be provided by the assessment of the role of BAG3 in response to therapy in melanoma cells. This in turn will lead to a rational basis for combination strategies that will include BAG3 silencing/inhibition aimed at circumventing resistance.

## RESULTS

### BAG3 protein is highly expressed in melanoma metastasis carrying BRAFV600E mutation and sustains BRAFV600E levels in A375 melanoma cells

**Figure 1:**
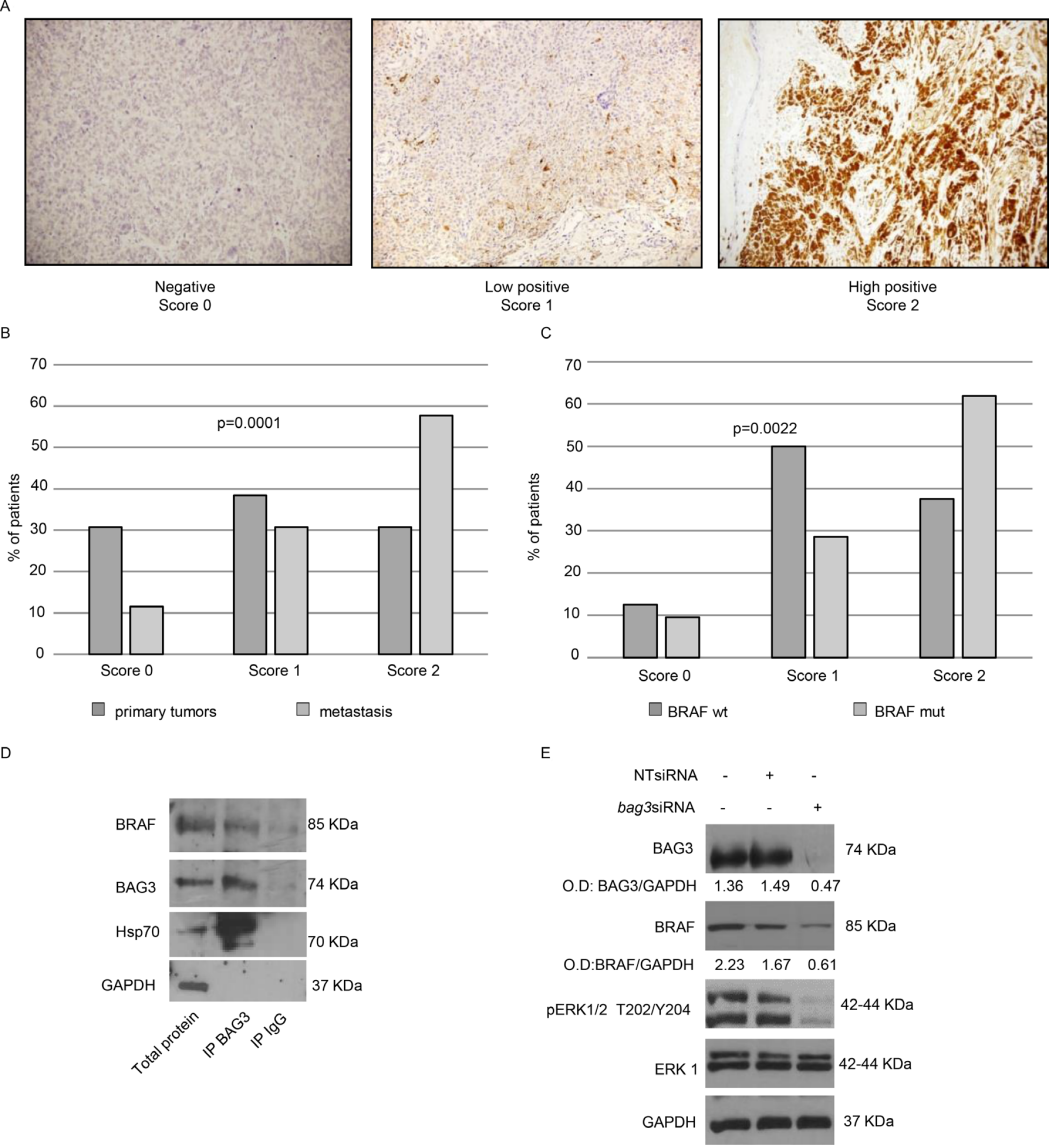
Analysis of BAG3 expression in human melanoma's metastases and its functional correlation with BRAFV600E Representative images of BAG3 negative (score 0), BAG3 low positive (score 1) and BAG3 high positive (score 2) metastatic melanoma samples stained using a monoclonal anti-BAG3 antibody revealed by using a biotinylated secondary antibody. Sections were counterstained with hematoxylin. (**A**) mut, mutation; WT, wild type. Fisher exact test was calculated by using 2 × 3 contingency tables. (**B**, **C**) A375 extracts were immunoprecipitated with an anti-BAG3 monoclonal antibody and immune complexes were then immunoblotted with antibodies recognizing BRAF, BAG3, Hsp70, or GAPDH as indicated. Immunoprecipitation with mouse IgGs was used as negative control (**D**) BAG3 down-modulation reduces levels of BRAF protein and affects ERK phosphorylation in A375 cells. A375 cells were transfected twice consecutively with a BAG3-specific or a non-targeting (NT) siRNA (200 nM), with the second transfection time being 72 hrs after the first one. After 120 hrs collected from the first transfection cells were analysed by western blot using anti-BAG3 polyclonal, anti-BRAF, anti-pERK and anti-ERK1 antibodies. Anti-GAPDH antibody was used as loading control. The levels of BAG3, BRAF were quantified by densitometry and normalized to GAPDH (O.D. BAG3/O.D. GAPDH; O.D. BRAF/O.D. GAPDH) (**E**) *p < 0.05 > 0.01; **p < 0.01 > 0.001.

BAG3 protein has been described for its anti-apoptotic role in melanoma cells [17] and its expression in melanoma metastatic lymph nodes was correlated to the aggressiveness of the tumour [11]. These pieces of evidence prompted us to deeper analyse a possible involvement of BAG3 in melanoma tumour development. To this end, we analysed BAG3 expression in a series of tissue samples from tumours and metastasis coming from 41 patients with advanced malignant melanoma, by immunohistochemistry (IHC), using an anti-BAG3 monoclonal antibody (AC-1). Intensity and distribution of immunostaining was used to assign to the BAG3 signal a score from 0 to 2. In particular, tumour tissue samples showing high positivity were classified with a score 2; those samples were characterized by a strong to moderate staining and a homogeneous distribution of positivity within tumour cells. Conversely, scores 1 or 0 were assigned when the BAG3 immunostaining was weak or absent, respectively (Figure 1A). In our series, we identified a subgroup composed by 26 patients for whom we had information about BAG3 staining in primary tumours and metastasis. As shown in Figure 1B, our analysis revealed that BAG3 expression is significantly enhanced in metastatic lesions as compared to primary tumours in this subgroup of patients. Indeed, more than the 55% of patients’ metastasis were classified with score 2 while only 10% resulted negative. (Fisher exact test *p* = 0.0001). Furthermore, in 10 of these patients we observed BAG3 positivity within the tumour tissue increased in metastatic sample in respect to the primary tumour. These data suggest a potential role of the anti-apoptotic BAG3 protein in maintaining metastatic melanoma cell survival and in sustaining tumour development.

In melanoma disease, approximately 50–60% of tumours contain a mutation in the gene that encodes BRAF that leads to constitutive activation of downstream signaling in the MAP kinase pathway. In a previous report [10], we did not observe any significant changes in BAG3 positivity distribution between BRAF WT and BRAF mutated melanoma samples of primary tumours. In our series we obtained similar results (data not shown), and in addition, we analysed BAG3 expression in metastatic samples of 21 patients carrying BRAFV600E mutation compared to that of 8 patients with a wild-type *BRAF* gene. We observed that high BAG3 expression appears to be significantly more frequent in BRAF mutated metastatic specimens as shown in Figure 1C (Fisher exact test *p* = 0.0022). This result demonstrated that BAG3 protein is highly expressed in melanoma metastatic cells carrying BRAFV600E mutation.

Evidences from a recent report have demonstrated that BAG3 protein sustains anaplastic thyroid carcinoma (ATC) growth either *in vitro* or *in vivo*. The molecular mechanism relies on BAG3 binding to BRAF, protecting the latter from proteasome-dependent degradation mediated by Hsp-70 [18]. Analogously to cutaneous malignant melanoma, ATC is characterized by the BRAFV600E mutation activating the kinase domain, which in turn strongly sustains the proliferative and oncogenic characteristics of these human tumour cells, mainly via ERK kinase [19]. Thus, we firstly investigated if BAG3 and BRAF protein interacts in A375 melanoma cells that harbour BRAFV600E mutation. To verify BAG3/BRAF interaction we performed a co-IP experiment and we found that BRAF protein was co- immunoprecipitated with BAG3 using a BAG3-specific antibody. Moreover, Hsp70 was found to co-immunoprecipitate with both BAG3 and BRAF (Figure 1D), as reported for ATC [18]. Next, we sought to determine if BAG3 silencing could accelerate BRAF degradation, thus lowering its total levels in the cells. As illustrated in Figure 1E, cell treatment with *bag3*siRNA resulted in reduction of BRAF intracellular levels, compared with BRAF levels in control and NT siRNA-treated cells and interestingly reduction of BRAF levels resulted in the lack of ERK protein phosphorylation. Those pieces of evidences suggest that BAG3 protein is involved in one of the major mechanism that sustain melanoma cells growth *id est* the axis BRAF/MEK/ERK.

### A375 melanoma cells resistance to BRAF inhibitor Vemurafenib is overcome by BAG3 silencing

In recent years, specific inhibitors of BRAF/MEK/ERK pathway alone or in combination have been used in patients with advanced melanoma and, although resistance to a combined therapy with BRAF inhibitor and MEK inhibitor resulted in a prolonged patients’ survival compared to treatment with the single agents, resistance remains a significant problem [20]. Mechanisms of cancer cells resistance to Vemurafenib can be established through two major mechanisms: ERK signaling activation in presence of the BRAF inhibitor and activation of parallel pro-survival pathways [7]. The first one is very frequently due to BRAF amplification, NRAS mutations, BRAF splice variants, all promoting dimerization of mutant BRAF with CRAF or wild type BRAF [21]. The latter refers to activation of PI3K/mTOR signaling cascade.

It was previously demonstrated that BAG3 inactivation or overexpression results in induction or inhibition, respectively, of both the spontaneous and chemotherapy-induced apoptosis [8]. As BAG3 protein is involved in sustaining BRAF levels and ERK phosphorylation in A375 melanoma cells, we sought to verify if BAG3 silencing can affect the response of those cells to prolonged Vemurafenib treatment.

**Figure 2:**
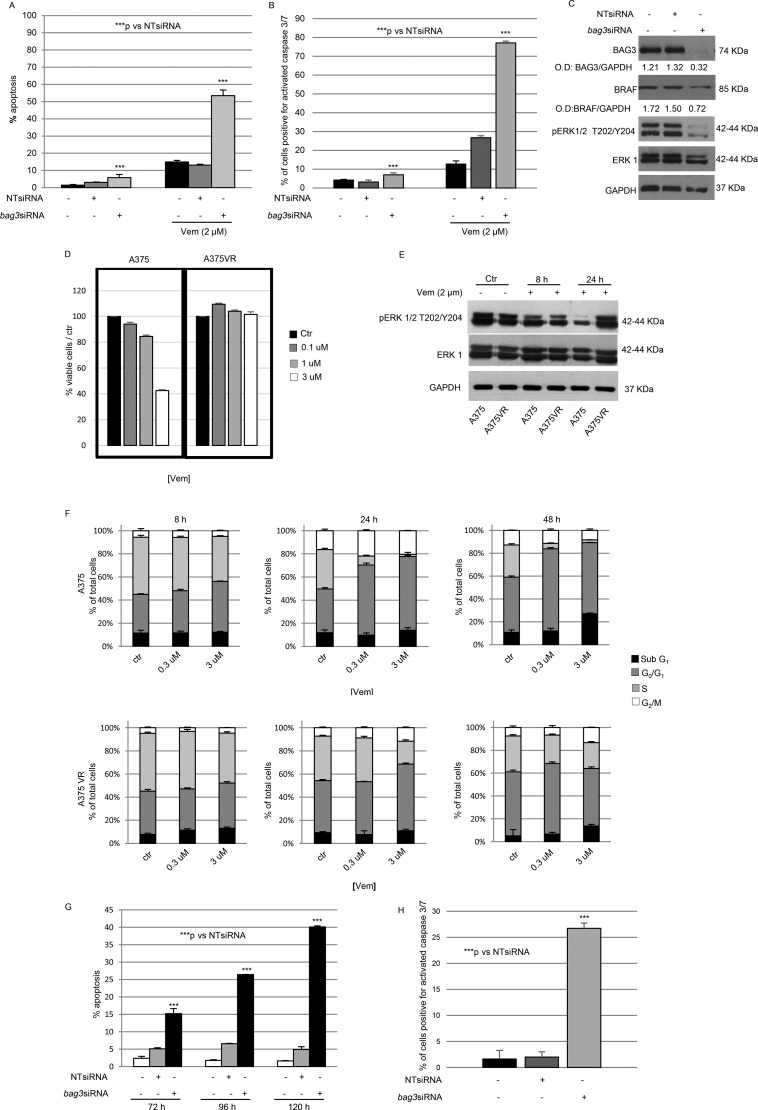
Down-regulation of BAG3 sensitizes A375 cell line to Vemurafenib via ERK pathway A375 cells were transfected twice consecutively with a BAG3-specific or a non-targeting (NT) siRNA (200 nM), with the second transfection time being 72 hrs after the first one and they were treated with Vemurafenib (2 μM). After 120 hrs from the first transfection, cells were collected and labelled with propidium iodide and analysed by flow cytometry. The percentage of cells in the sub-diploid apoptotic region was quantified for each condition. Graph depicts mean percentage of Sub G0/G1 cells (± SD). (**A**) A375 cells were treated with a BAG3-specific or a non-targeting (NT) siRNA (200 nM) as previously described and stained with 5 μM CellEvent^™^ Caspase-3/-7 Green detection reagent for 30 min at 37°C and analysed by flow cytometry. Data are presented as the mean ± SD of three independent determinations (**B**) BAG3 down-modulation reduces levels of BRAF protein and affects ERK phosphorylation in A375 cells. A375 cells were transfected as previously described and after 120h total protein extracts were analysed by western blot using anti-BAG3 polyclonal, anti-BRAF, anti-pERK and anti-ERK1 antibodies. Anti-GAPDH antibody was used as loading control. The levels of BAG3, BRAF were quantified by densitometry and normalized to GAPDH (O.D. BAG3/O.D. GAPDH; O.D. BRAF/O.D. GAPDH) (**C**) A375 Acquire resistance to Vemurafenib (PLX4032) after long-term drug treatment. We cultured BRAF mutant melanoma cells (A375) in increasing concentration (up to 2 μM) of the B-RAF inhibitor Vemurafenib (PLX4032). After 2 months, we isolated a resistant cell line (A375VR) that was less sensitive to PLX4032 than the parental cell line. Parental and resistant cells were grown in the presence of indicated doses of Vemurafenib for 120 hrs. Relative cell viability was assessed by MTT assay (**D**) Resistant cells bypass G1/S arrest induced by PLX4032. A375 and A375VR were treated with 2 μM Vemurafenib for 8 and 24 hrs. Then the levels of phosphorylated ERK (pERK) and ERK 1 were analysed by western blot using anti-pERK and anti-ERK antibodies. GAPDH was used as loading control (**E**) A375 and A375VR were treated with different doses of Vemurafenib for indicated time. Cells were harvested and stained with propidium iodide for cell-cycle analysis (**F**) Down-regulation of BAG3 re-sensitizes A375VR cell lines to Vemurafenib. A375VR cells were transfected with a BAG3-specific or a NT siRNA (200 nM). After 24 hrs they were treated with 2 μM Vemurafenib and after 72, 96, and 120 hrs cells were collected, labelled with propidium iodide and analysed by flow cytometry. The percentage of cells in the sub-diploid apoptotic region was quantified for each condition. Graph depicts mean percentage of Sub G0/G1 cells (± SD) (**G**) A375VR cells were treated with a BAG3-specific or a non-targeting (NT) siRNA (200 nM) as previously described and stained with 5 μM CellEvent™ Caspase-3/-7 Green detection reagent for 30 min at 37°C and analysed by flow cytometry. Data are presented as the mean ± SD of three independent determinations (**H**) *p < 0.05 > 0.01; **p < 0.01 > 0.001; ***p < 0.001.

To this end, A375 cells were transiently transfected with *bag3*siRNA or non-targeting siRNA (NT-siRNA) at a final concentration of 200 nM and treated them with 2 μM Vemurafenib for 120 hours. As shown in Figure 2A, the levels of hypo-diploid nuclei induced by Vemurafenib were significantly increased in cells where BAG3 was silenced. We also analysed in the same experimental settings cleaved caspase-3/7 contents and we confirmed that interfering on BAG3 protein expression sensitizes to apoptosis melanoma cells subjected to a prolonged treatment with Vemurafenib (Figure 2B).

BRAF mutated protein has a basal kinase activity 10fold higher than BRAF wildtype counterpart, resulting in hyper-activation of the MEK/ERK pathways [22]. Interestingly, extended Vemurafenib treatment induced a rebound of phospho-ERK1/2 as a first sign of resistance establishment [23]. We wanted to verify levels of BRAF end phospho-ERK1/2 in BAG3 silenced cells after this prolonged treatment with Vemurafenib (2 μM for 120 hours). As shown in Figure 2C we observed that BAG3 down-modulation during BRAF inhibitor treatment resulted in the loss of ERK phosphorylation thus suggesting that BAG3 can sustain ERK activation in presence of BRAF inhibitor. Interestingly, also BRAF levels, as observed in untreated cells, resulted to be down-modulated suggesting that resistance pathways due to BRAF gene gain or amplification could be sensitive to BAG3 silencing. This aberration was detected in patients treated with BRAF inhibitor alone but also in that treated in combination with a MEK inhibitor [24]. Furthermore, it was previously demonstrated that different levels of expression of the BRAFV600E modulate melanoma sensitivity to Vemurafenib [25].

In order to better analyse the role of BAG3 protein in Vemurafenib-resistant cells we cultured A375 cells with increasing concentrations (0.02 to 2 μM) of the BRAF inhibitor and cells able to grow in presence of 2 μM of Vemurafenib emerged after ∼2 months of culture. We obtained a cell line A375VR (A375 Vemurafenib Resistant). Notably, these cells displayed larger cell size and elongated morphology (data not shown). As shown in Figure 2D while A375 parental cell line displayed more than 50% of mortality after 120 hours of treatment with Vemurafenib at a 3 μM concentration, A375VR did not lose viability in respect to control cells.

To further confirm the establishment of resistance in A375VR, we tested the effect of Vemurafenib at the concentration of 2 μM, a dose at which the resistant cells were commonly cultured, on ERK phosphorylation in resistant cells compared with their parental counterpart. Vemurafenib treatment of parental cells led to inhibition of phospho- ERK1/2 after 24 hours, while resistant cells were not responsive to BRAF inhibitor in terms of phospho-ERK1/2 down-regulation. Additionally, we analysed cell-cycle profiles of parental and resistant cells treated with Vemurafenib at two different doses (0.3 and 3 μM) at 8, 24, and 48 hours (Figure 2F). Short-term (8 hours) treatment of parental cells with Vemurafenib had limited effects on the cell-cycle profile in A375 cells, whereas prolonged treatment with Vemurafenib (24–48 hours) led to strong reduction of S-phase cells at all doses and the accumulation of sub G1 cells at the dose of 3 μM after 48 hours. On the other hand, resistant cells were capable to overcome Vemurafenib- induced cytostatic effect accompanied by cell death.

To investigate BAG3 protein involvement in acquired resistance to Vemurafenib, we assessed the effect on cell apoptosis of the *bag3*siRNA on A375VR. For this purpose, we treated A375VR cells with a specific siRNA for BAG3 or NT-siRNA while continuously exposed to Vemurafenib 2 μM and analysed the cells for hypo-diploid nuclei contents at 72, 96, and 120 hours. In Figure 2G, A375VR cells displayed a significant re-sensitization to Vemurafenib when treated with a specific *bag3*siRNA after 72 hours; rate of apoptosis reached 40% after 120 hours of continuous exposure to BRAF-inhibitor and *bag3*siRNA. Since the maximal apoptotic effect was found at 120 hours, this time point was chosen for subsequent experiments. Indeed, we also observed in the same experimental setting a significant increase in appearance of cleaved caspase-3/7 in cells were BAG3 was silenced, as shown in Figure 2H.

Those findings indicate that in A375 melanoma cells Vemurafenib Resistant can be sensitized to apoptosis by interfering with BAG3 protein levels.

### BAG3 down-modulation restores A375VR sensitivity to Vemurafenib acting on ERK pathway but also on parallel survival pathways

To identify pathways implicated in acquired resistance to Vemurafenib, we plated A375VR at low density and 5 different clones were selected: A375VR#5, A375VR#6, A375VR#7, A375VR#8, and A375VR#9. Selected clones were continuously kept in culture in presence of 2 μM Vemurafenib.

**Figure 3:**
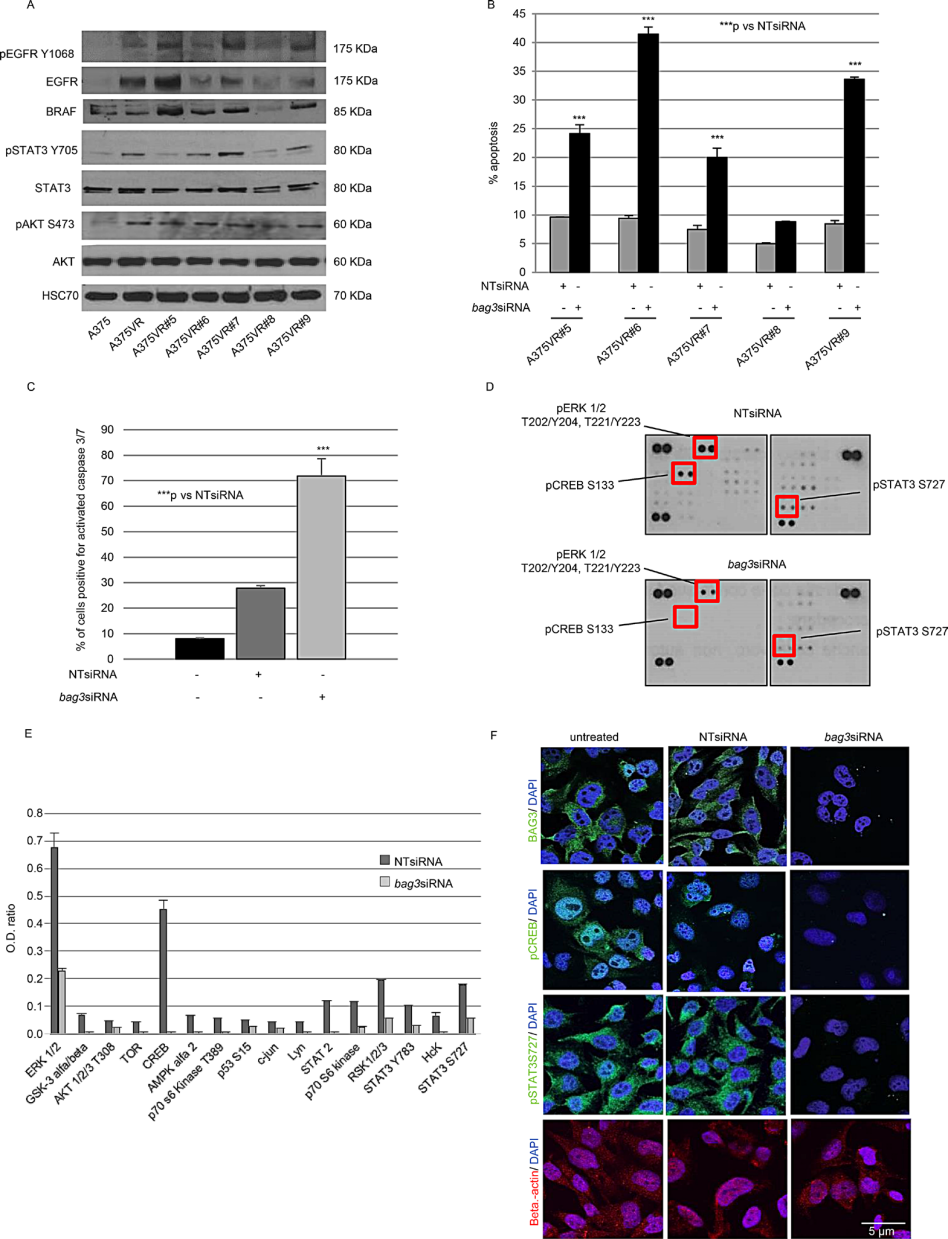
Analysis of BRAF inhibitor resistance pathways affected by BA63 levels in melanoma cells Five resistant clones were generated from A375VR. Western Blots for phosphorylated EGFR (pEGFR), EGFR, BRAF, phosphorylated AKT (pAKT), AKT, phosphorylated STAT3 (pSTAT3 Y705), STAT3 and Hsc70 (loading control) in A375, A375VR, A375VR#5, A375VR#6, A375VR#7, A375VR#8, and A375VR#9 cells (**A**). Down-regulation of BAG3 sensitizes A375VR#5, A375VR#6, A375VR#7, A375VR#8, and A375VR#9 cells lines to Vemurafenib. A375VR#5, A375VR#6, A375VR#7, A375VR#8, and A375VR#9 cells were transfected with a BAG3-specific or a NT siRNA (200 nM) (see figure legend 1D). After 120 hrs, cells were collected, labelled with propidium iodide, and analysed by flow cytometry. The percentage of cells in the sub-diploid apoptotic region was quantified for each condition. The graphic depicts mean percentage of Sub G0/G1 cells (± SD) (**B**). A375VR#6 cells were treated with a BAG3-specific or a non-targeting (NT) siRNA (200 nM) as previously described and stained with 5 μM CellEvent™ Caspase-3/-7 Green detection reagent for 30 min at 37°C and analysed by flow cytometry. Data are presented as the mean ± SD of three independent determinations (**C**). BAG3 silencing induces down-modulation of many crucial proteins involved in cell survival in A375VR#6 resistant clone. A375VR#6 cells were transfected with a BAG3-specific or a NT siRNA (200 nM), as previously described. After 120 hrs cells were collected and analysed with human phospho-array kit following manufacturing instructions (**D, E**). BAG3 silencing induces apoptosis through pSTAT3 and pCREB down-modulation in A375VR#6 clone. Analysis by confocal microscopy using anti-phosphorylated STAT3 (pSTAT3-S727), anti-phosphorylated CREB (pCREB), anti-BAG3, anti-BRAF and anti-cleaved caspase 3 antibodies. Anti-GAPDH and anti-beta-actin antibodies were utilized as control (**F**). *p < 0.05 > 0.01; **p < 0.01 > 0.001; ***p < 0.001.

As already reported, the pathway activated by the EGF receptor (EGFR) plays a crucial role in resistance of melanoma cells to Vemurafenib [26]. To further confirm these data, phosphorylated EGFR (pEGFR) and EGRF protein levels were analysed by Western Blotting in selected clones and compared with those in A375 and A375VR. As shown in Figure 3B, A375VR were characterized by increased levels of both pEGFR and EGFR proteins in comparison with those detected in A375. On the other end, each clone was characterized by different levels of pEGFR and EGFR proteins. We have also shown that the phosphorylation of AKT, an EGFR downstream effector, was enhanced in resistant cells and in clones. Our analysis confirmed that phosphorylation of some signal transducers and transcription activators was consistently increased. Such an increase was also evident for STAT3 phosphorylation in resistant cells and clones; on this regard, the proteins belonging to the STAT family of transcription factors, they are activated by cytokines and growth factors receptors and led to cancer progression [27] (Figure 3A).

To further identify mechanisms that were implicated in driving resistance in our experimental model, we tested the expression of BRAF in Vemurafenib resistant sub clones by Western Blotting. As shown in Figure 3A, there was no difference in BRAF protein expression between A375VR cell line and their parental counterpart, but all five A375VR sub clones expressed different levels of BRAF mutated protein.

To investigate the mechanisms by which the increased expression levels of pEGFR, EGFR, pAKT, pSTAT3, and BRAF could influence response of Vemurafenib-resistant clones to BAG3 down-modulation, we transfected the five A375VR sub clones with *bag3*siRNA or NTsiRNA while continuously culturing them with 2 μM Vemurafenib, as previously described for A375VR. After 120 hours from transfection, we evaluated the percentage of cells with hypodiploid nuclei by propidium iodide staining. BAG3 down-modulation was found to be able to re-sensitize the different clones, though the degree of such effect again varied between the different clones.

In particular, A375VR#6 was the clone more susceptible to BAG3 down-modulation, reaching a rate of apoptosis of 42.5% after 120 hours from transfection (Figure 3B). The induction of apoptosis in A375VR#6 subjected to BAG3 silencing was further confirmed by the appearance of cleaved caspase-3, as shown in Figure 3C. BAG3 silencing in A375VR#6 resulted also in the reduction of BRAF levels as demonstrated in the parental cell line (data not shown).

A375VR#6 clone was used to deeper investigate the molecular mechanisms through which BAG3 silencing could restore sensitivity to Vemurafenib in melanoma cells with acquired resistance to this inhibitor.

We conducted phosphoprotein-array analysis to identify any pathway that may be deregulated in the resistant clone after treatment with a specific agent able to knockdown BAG3 protein expression. Therefore, A375VR#6 cells were treated with *bag3*siRNA or NTsiRNA as previously described and, after 120 hours cells were collected, lysed, and analysed with human phosphoprotein array. We observed down-modulated phosphorylation of the ERK protein, after BAG3 protein silencing in the resistant clone. Furthermore, we observed that RSK and the transcription factors CREB, and STAT3/2, three downstream target of pERK were down-modulated as well (Figure 3D–3E). It is of note that, in recent papers it was demonstrated that molecules able to interfere with STAT3 are able to overcome Vemurafenib resistance in melanoma cells [27] and that hyperactivation of CREB confers acquired resistance to to BRAFV600E inhibition in melanoma via upregulation of AEBP1 expression and consequent activation of NF-κB [28].

Targets of the PI3K pathway were also found to be significantly dephosphorylated in cells where BAG3 was silenced such as AKT, mTOR and the p70S6K that also were found to be interesting targets to overcome acquired resistance to to BRAFV600E inhibition in melanoma [29].

Interestingly, also Hck and Lyn of the family of Src Tyrosine Kinases were down-phosphorilated when BAG3 expression is inhibited; those proteins act upstream of ERK kinase and result activated upon aberrant expression and/or activity of EGFR. Inhibition of EGFR or Src Tyrosine Kinases were also found to be effective in overcome BRAF inhibitor resistance in melanoma cells [30].

We also confirmed that transcription factors CREB and STAT3 phosphorylation was decreased in the A375VR#6 cell line by immunofluorescence upon transfection with a specific *bag3siRNA* (Figure 3F).

Alltogheter, our data show that BAG3 can lower several survival pathways hyper-activated during the acquisition of the resistance.

## DISCUSSION

The increased knowledge about the molecular mechanisms underlying the pathogenesis of cutaneous melanoma has led to the development of a personalized approach for the treatment of melanoma [31]. Treatment of BRAF-mutant metastatic melanoma with mitogen-activated protein kinase (MAPK) pathway targeted therapies (BRAF/MEK inhibitors) has improved outcomes and revolutionized disease management for patients with advanced stage disease [7]. However, the clinical experience with BRAF inhibitors and in particular with Vemurafenib has also shown that the efficacy of long-term treatment for patients with melanoma is hampered by the development of acquired drug resistance [6, 32]. Therefore, it is important to determine whether BRAFV600E- mutant melanoma cells with acquired resistance to Vemurafenib could be re-sensitized to the treatment.

BAG3 is an anti-apoptotic protein that has been shown to sustain cell survival in a variety of tumour types, including melanoma [9, 12, 13, 14, 15, 16]. BAG3 down-regulation appears to indeed induce cell apoptosis and impair tumour growth in melanoma - both *in vitro* and *in vivo* [17] - suggesting that it may represent a novel target for tumour therapy. It has been indicated that the role of BAG3 in melanoma is due to its anti-apoptotic properties; in fact, this protein has been shown to protect melanoma cells from death through the interaction with apoptosis-regulating proteins, such as the IKKγ subunit of the NF-κB-activating complex IKK [17]. Furthermore, BAG3 expression was reported to be associated with melanoma progression [10]; more recently, an interesting correlation between BAG3 protein expression and prognosis in patients affected by metastatic melanoma with positive lymph nodes has been described [11].

Results shown in this report describe that BAG3 is highly expressed in in the majority of metastatic melanoma carrying the BRAFV600E mutation and, therefore, this protein could be a possible therapy target. Moreover, in an *in vitro* model of acquired resistance to Vemurafenib, we have demonstrated that BAG3 silencing was able to restore sensitivity to the BRAF inhibitor and that the mechanism responsible for such a re-sensitization seems to be effective on more than one survival pathway responsible of the acquired resistance. We can summarize that BAG3 silencing is able to significantly impact on one of the major feature of BRAF inhibitor resistance that is a persistent ERK phosphorylation, this impact on the downstream target as RSKs, CREB and STAT3. Interestingly, BAG3 down-modulation impacts also on Lyn and Hck Tyrosine Kinases that work upstream in respect to ERK and are found hyper-phosphorylated upon aberrant expression and/or activity of EGFR. It is of note that, other proteins, containing the Src module were found to interact with BAG3 via its proline rich domain [33]. Furthermore, BAG3 is also involved in the PI3K/AKT/mTOR survival pathway; indeed, BAG3 sustains levels of AKT and its downstream targets as previously reported [34]. Recently, has also been described an inhibitor of Hsp70 able to disrupt BAG3/Hsp70 complex [33] that acts via the BAG domain of the BAG3 protein. We believe that a part of the observations we reported in this paper can be ascribed to the Hsp70- mediated activities of BAG3 protein, such for instance the effects on BRAF and AKT. However, the activity on the other targets may be obtained through the other functional domains of BAG3 such as the WW domain, the proline rich or the IPV motif. Thus, additional studies and the design of molecules that can selective bind to this portions of the protein can represent a valid tool to selectively control and inhibit survival pathways in resistant cells.

In conclusion, our data strongly support the idea that BAG3 can represent a valid target in the treatment of BRAF inhibitor resistant metastatic melanomas.

## MATERIALS AND METHODS

### Cell cultures and reagents

The melanoma cancer cell line A375 was obtained from the American Type Culture Collection (ATCC, Manassas, VA, USA) and cultured in DMEM (Dulbecco's modified Eagle's medium) supplemented with 10% fetal bovine serum (FBS). The media for culturing the above cell line were purchased from Lonza (Bergamo, Italy) and supplemented with 100 U/mL penicillin and 2 g/mL streptomycin (Sigma-Aldrich Corp). A375 cells were cultured in increasing concentrations of PLX4032 (from 0.1–2 μM) to generate resistant cell line (A375VR). To generate the resistant clones A375VR#5, A375VR#6, A375VR#7, A375VR#8 and A375VR#9 the resistant cell line (A375VR) was plateted at the density of 1 cell/well in a 96 well plate. Resistant lines were maintained in the continuous presence of 2 μM PLX4032, supplemented every 72 hr.

Vemurafenib was obtained from Selleckchem (Houston, TX, USA).

### Immunohistochemistry

Four-μm thick sections of each tissue, mounted on poly-L-lysine-coated glass slides, were analysed by immunohistochemistry (IHC) using the anti-BAG3 mAb AC-1 (BIOUNIVERSA s.r.l., SA, Italy). IHC protocol included deparaffination in bioclear, rehydration through descending degrees of alcohol up to water, incubation with 3% hydrogen peroxidase for 5 minutes to inactivate endogenous peroxidases, non enzymatic antigen retrieval in CC1 buffer (Ventana Medical System), pH 8.0, for 36 minutes at 95°C. After rinsing with phosphate-buffered saline (PBS 1×), samples were blocked with 5% fetal bovine serum in 0.1% PBS/BSA and then incubated for 1 hour at room temperature with the mAb in saturating conditions. The standard streptavidin-biotin linked horseradish peroxidase technique was then performed, and 3,3′-diaminobenzidine was used as a substrate chromogen solution for the development of peroxidase activity. Finally, the sections were counterstained with hematoxylin; slides were then coverslipped using a synthetic mounting medium. For immunohistochemistry, scoring was performed by at least two investigators (in very few borderline cases, classification of BAG3 staining required additional investigators and was based on the consistency of the majority of them).

### Patient samples

Tumour samples were obtained from a consecutively-collected series of unselected patients who underwent surgical resection of metastatic malignant melanoma at both the Local Health Unit 1 (ASL1) and Azienda Ospedaliero Universitaria (AOU), Sassari (Italy). Patients were informed about aims and limits of the study and gave their written informed consent before tissue samples were collected. The study was reviewed and approved by the ethical review boards of both Local Health Unit 1 (Azienda Sanitaria Locale 1; ASL1) and University of Sassari.

### Western blot

Cells were harvested and lysed in a buffer containing 20 mM HEPES (pH 7.5), 150 mM NaCl, 0.1% Triton (TNN buffer) supplemented with a protease inhibitors cocktail (Sigma), and subjected to 3 cycles of freeze-and-thawing. Lysates were then centrifuged for 20 min at 15,000 × g and stored at −80°C. Protein amount was determined by Bradford assay (Bio-Rad, Hercules, CA) and 30 μg of total protein were separated on 10% SDS-PAGE gels and electrophoretically transferred to nitrocellulose membrane. Nitrocellulose blots were blocked with 10% nonfat dry milk in TBST buffer (20 mM Tris-HCl at pH 7.4, 500 mM NaCl and 0.01% Tween), and incubated with primary antibodies in TBST containing 5% non-fat dry milk overnight at 4°C. Immunoreactivity was detected by sequential incubation with horseradish peroxidase-conjugated secondary antibodies and ECL detection reagents (Amersham Life Sciences Inc., Arlington Heights, IL, U.S.). Signal detection was performed using ImageQuant^™^ LAS 4000 (GE Healthcare, U.S.).

### Apoptosis

Cells were seeded in 24-well plates (1 × 10^4^ cells per well) and treated with 2 μM of Vemurafenib and/or transfected with BAG3 small-interfering RNA (siRNA) or NonTargeted siRNA (NTsiRNA). At the end of treatment, the percentage of sub-G0/G1 cells was analysed via propidium iodide incorporation into permeabilized cells, and flow cytometry was performed as previously described [17]. Each experimental point was performed in triplicate, and the data reported are the mean of at least three independent experiments. Error bars depict Standard Deviation (SD).

For the quantification of caspase-3/7 activity, cells were labeled with 500 nM CellEvent caspase-3/7 green detection reagent (Life Technologies) for 30 minutes at 37°C. A total of 10,000 stained cells per sample were acquired and analyzed in a FACSVerse flow cytometer by using FACSSuite software (Becton Dickinson).

### siRNAs and transfections

*bag3* siRNA b (5′-ATCGAAGAGTATTTGA CCAAA-3′) and NT siRNA (5′-CAGUCGCGUUUGCG ACUGG-3′) were synthesized by MWG Biotech (Ebersberg, Germany). Cells were transfected with siRNAs at a final concentration of 200 nM usingTransFectin (Bio-Rad Laboratories, Inc., Hercules, CA, USA). Transfection efficiency was evaluated in each experiment by western blot analysis.

### Co-immunoprecipitation

For immunoprecipitation of BAG3 protein the anti-BAG3 mAb AC-2 was coupled to Dynabeads (Invitrogen) following the manufacturer's instructions. Briefly, 500 × g of cell extracts were immunoprecipitated at 4°C overnight and then analysed by Western blot using a rabbit anti-BAG3 polyclonal primary antibody, anti-BRAF antibody, anti-HSC70 antibody, and anti-GAPDH antibody.

### Cell viability and cell-cycle analysis

Cells were synchronized by holding at confluence for 2 days, which arrests cells in G0/G1 phase of the cell cycle (DAI et al., 2007). Synchronized cells were plated at a cell density of 5 × 10^3^ cells/cm^2^ and after 2 h were treated with indicated concentrations of Vemurafenib (see figure legends). Cell viability was measured by MTT ([3-(4,5-dimethylthiazol-2-yl)-2,5-diphenyl tetrazolium bromide]) (M2128) assay. Cell-cycle analysis was performed after incubation of cells with PI solution (2.5 mg/ml propidium iodide, 0.75 M sodium citrate pH 8.0, 0.1% Triton). Fluorescence intensity was measured by flow cytometry (FACScan Becton Dickinson, BD, Franklin Lakes, NJ, USA). For each sample, 10,000 events were recorded and histograms of red fluorescence versus counts were generated to evaluate percentages of cells in each phase of the cell cycle. The proportion of cells in each phase was calculated using ModFit LT software (BD).

### Indirect immunofluorescence

Cells were cultured on coverslips in six-well plates to 60–70% confluence and transfected with 200 nM bag3siRNA or NTsiRNA. After 120 hours of transfection, coverslips were washed in PBS 1× and fixed in 3.7% formaldehyde in PBS1X for 30 min at room temperature, and then incubated for 5 min with PBS 1× 0.1 M glycine. After washing, coverslips were permeabilized with 0.1% Triton X-100 for 5 min, washed again and incubated with blocking solution (5% normal goat serum in PBS 1×) for 1h at room temperature. Following incubation with 3 mg/ml of anti-BAG3 mouse monoclonal antibody AC-2 at room temperature for 1 hour a 1:800 dilution of anti-pCREB, 1: 400 of anti-pSTAT3S727 and 1: 100 of anti- beta- actin antibody, coverslips were washed three times with PBS 1×. After incubation with a 1: 500 dilution of goat anti-mouse or anti-rabbit IgG DyLigth 488-conjugated antibodies (Jackson ImmunoResearch, West Grove, PA, USA) and a 1: 500 dilution of goat anti-mouse IgG DyLigth 649-conjugated antibodies (Jackson ImmunoResearch) at room temperature for 45 min, coverslips were again washed for three times in PBS and then in distilled water. The coverslips were then mounted on a slide with interspaces containing 47% (v/v) glycerol. Samples were analysed using a confocal laser scanning microscope (Leica SP5, Leica Microsystems, Wetzlar, Germany). Images were acquired in sequential scan mode by using the same acquisitions parameters (laser intensities, gain photomultipliers, pinhole aperture, objective 63×, zoom 1.1) when comparing experimental and control material. For production of figures, brightness and contrast of images were adjusted by taking care to leave a light cellular fluorescence background for visual appreciation of the lowest fluorescence intensity features and to help comparison among the different experimental groups. Final figures were assembled using Adobe Photoshop 7 and Adobe Illustrator 10 (Adobe systems incorporated, San Jose, CA, USA). Leica Confocal Software and ImageJ (Leica Microsystems, Wetzlar, Germany) were used for data analysis.

### Antibodies and kit

Anti-BAG3 rabbit polyclonal and murine monoclonal antibodies were obtained from BIOUNIVERSA s.r.l., SA, Italy. The rabbit polyclonal anti-BAG3 was raised against the full-length BAG3 recombinant protein. AC2 interacts with a portion of BAG3 protein (from aminoacid 385 to aminoacid 399) not overlapping the BAG domain. This was produced in endotoxin free conditions by Nanotools (Teningen, DE). The anti-BAG3 monoclonal murine clone AC-1 used for staining specifically interacts with the BAG3 region from aminoacid 18 to aminoacid 33. Antibodies recognizing ERK1 (sc-94), BRAF (sc-166), STAT3 (sc-80109), GAPDH (sc-32233), Hsc-70 (sc-7298), and Beta-actin (sc-477789) were obtained from Santa Cruz Biotechnology, Inc. (Santa Cruz, CA, USA); p-ERK1/2 (Thr202/Thr404; #9101), pSTAT3 (Ser727; #9136), pCREB (Ser133; #9198), ), pAKT (Ser473; #9271), AKT (#9272), EGFR (#4267) cleaved caspase 3 (Asp175; #9661) from Cell Signaling Technology, Inc. (Danvers, MA, USA), pEGFR (Y1068; #04–339) from Millipore (Darmstadt, Germany). Mouse IgG isotype control (ABIN 398652) was obtained from Antibodies-online.

Human Phospho-Kinase array kit (ARY003B) was obtained from R&D system (Minneapolis, USA). Levels of phosphorylated proteins (300 μg per sample) were analyzed in cell lysates according to the protocol provided by the manufacturer. The array was analyzed and quantified by using ImageJ software (CA, USA). Pixels in each spot were calculated and subtracted to that of background, then the value obtained was normalized on the value obtained in reference spots. For each phospho-protein we had 2 spots.

### Statistical analysis

Results are expressed as means ± standard deviation (SD). Data were analysed by Student's *t*-test using MedCalc statistical software version 13.3.3 (Ostend, Belgium). *P*-values from 0.01 to 0.05, from 0.001 to 0.01, or < 0.001 were considered significant (*), very significant (**) or highly significant (***), respectively.

## References

[1] Ferlay J, Soerjomataram I, Dikshit R, Eser S, Mathers C, Rebelo M, Parkin DM, Forman D, Bray F (2015). Cancer incidence and mortality worldwide: sources, methods and major patterns in GLOBOCAN 2012. Int J Cancer.

[2] Kim G, McKee AE, Ning YM, Hazarika M, Theoret M, Johnson JR, Xu QC, Tang S, Sridhara R, Jiang X, He K, Roscoe D, McGuinn WD (2014). FDA approval summary: vemurafenib for treatment of unresectable or metastatic melanoma with the BRAFV600E mutation. Clin Cancer Res.

[3] Jang S, Atkins MB (2013). Which drug, and when, for patients with BRAF-mutant melanoma?. Lancet Oncol.

[4] Nikolaou VA, Stratigos AJ, Flaherty KT, Tsao H (2012). Melanoma: new insights and new therapies. J Invest Dermatol.

[5] Kugel CH, Aplin AE (2014). Adaptive resistance to RAF inhibitors in melanoma. Pigment Cell Melanoma Res.

[6] Hartsough E, Shao Y, Aplin AE (2014). Resistance to RAF inhibitors revisited. Cancer Discov.

[7] Welsh SJ, Rizos H, Scolyer RA, Long GV (2016). Resistance to combination BRAF and MEK inhibition in metastatic melanoma: Where to next?. Eur J Cancer.

[8] Rosati A, Graziano V, De Laurenzi V, Pascale M, Turco MC (2011). BAG3: a multifaceted protein that regulates major cell pathways. Cell Death Dis.

[9] Rosati A, Basile A, Falco A, d'Avenia M, Festa M, Graziano V, De Laurenzi V, Arra C, Pascale M, Turco MC (2012). Role of BAG3 protein in leukemia cell survival and response to therapy. Biochim Biophys Acta.

[10] Franco R, Scognamiglio G, Salerno V, Sebastiani A, Cennamo G, Ascierto PA, Botti G, Turco MC, Rosati A (2012). Expression of the anti-apoptotic protein BAG3 human melanomas. J Invest Dermatol.

[11] Guerriero L, Chong K, Franco R, Rosati A, De Caro F, Capunzo M, Turco MC, Hoon DS (2014). BAG3 protein expression in melanoma metastatic lymph nodes correlates with patients' survival. Cell Death Dis.

[12] Chiappetta G, Basile A, Barbieri A, Falco A, Rosati A, Festa M, Pasquinelli R, Califano D, Palma G, Costanzo R, Barcaroli D, Capunzo M, Franco R (2014). The anti-apoptotic BAG3 protein is expressed in lung carcinomas and regulates small cell lung carcinoma (SCLC) tumor growth. Oncotarget.

[13] Rosati A, Bersani S, Tavano F, Dalla Pozza E, De Marco M, Palmieri M, De Laurenzi V, Franco R, Scognamiglio G, Palaia R, Fontana A, di Sebastiano P, Donadelli M (2012). Expression of the antiapoptotic protein BAG3 is a feature of pancreatic adenocarcinoma and its overexpression is associated with poorer survival. Am J Pathol.

[14] Festa M, Del Valle L, Khalili K, Franco R, Scognamiglio G, Graziano V, De Laurenzi V, Turco MC, Rosati A (2011). BAG3 protein is overexpressed in human glioblastoma and is a potential target for therapy. Am J Pathol.

[15] Chiappetta G, Ammirante M, Basile A, Rosati A, Festa M, Monaco M, Vuttariello E, Pasquinelli R, Arra C, Zerilli M, Todaro M, Stassi G, Pezzullo L (2007). The antiapoptotic protein BAG3 is expressed in thyroid carcinomas and modulates apoptosis mediated by tumor necrosis factor-related apoptosis-inducing ligand. J Clin Endocrinol Metab.

[16] Romano MF, Festa M, Petrella A, Rosati A, Pascale M, Bisogni R, Poggi V, Kohn EC, Venuta S, Turco MC, Leone A (2003). BAG3 protein regulates cell survival in childhood acute lymphoblastic leukemia cells. Cancer Biol Ther.

[17] Ammirante M, Rosati A, Arra C, Basile A, Falco A, Festa M, Pascale M, d'Avenia M, Marzullo L, Belisario MA, De Marco M, Barbieri A, Giudice A (2010). IKK{gamma} protein is a target of BAG3 regulatory activity in human tumor growth. Proc Natl Acad Sci USA.

[18] Chiappetta G, Basile A, Arra C, Califano D, Pasquinelli R, Barbieri A, De Simone V, Rea D, Giudice A, Pezzullo L, De Laurenzi V, Botti G, Losito S (2012). BAG3 down-modulation reduces anaplastic thyroid tumor growth by enhancing proteasome-mediated degradation of BRAF protein. J Clin Endocrinol Metab.

[19] Davies H, Bignell GR, Cox C, Stephens P, Edkins S, Clegg S, Teague J, Woffendin H, Garnett MJ, Bottomley W, Davis N, Dicks E, Ewing R (2002). Mutations of the BRAF gene in human cancer. Nature.

[20] Long GV, Weber GS, Infante JR, Kim KB, Daud A, Gonzalez R, Sosman JA, Hamid O, Schuchter L, Cebon J, Kefford RF, Lawrence D, Kudchadkar R (2016). Overall survival and durable responses in patients with BRAF V600-mutant metastatic melanoma receiving dabrafenib combined with trametinib. J Clin Oncol.

[21] McArthur GA (2015). Combination Therapies to Inhibit the RAF/MEK/ERK Pathway in Melanoma: We are not Done Yet. Front Oncol.

[22] Dhomen N, Marais R (2009). BRAF signalling and targeted therapies in melanoma. Hematol Oncol Clin North Am.

[23] Paraiso KH, Fedorenko IV, Cantini LP, Munko AC, Hall M, Sondak VK, Messina JL, Flaherty KT, Smalley KS (2010). Recovery of phospho-ERK activity allows melanoma cells to escape from BRAF inhibitor therapy. Br J Cancer.

[24] Manzano JL, Layos L, Bugés C, de los Llanos Gil M, Vila L, Martínez-Balibrea E, Martínez-Cardús A (2016). Resistant mechanisms to BRAF inhibitors in melanoma. Ann Transl Med.

[25] Shi H, Moriceau G, Kong X, Lee MK, Lee H, Koya RC, Ng C, Chodon T, Scolyer RA, Dahlman KB, Sosman JA, Kefford RF, Long GV (2012). Melanoma whole-exome sequencing identifies (V600E)B-RAF amplification-mediated acquired B-RAF inhibitor resistance. Nat Commun.

[26] Girotti MR, Pedersen M, Sanchez-Laorden B, Viros A, Turajlic S, Niculescu-Duvaz D, Zambon A, Sinclair J, Hayes A, Gore M, Lorigan P, Springer C, Larkin J (2013). Inhibiting EGF receptor or SRC family kinase signaling overcomes BRAF inhibitor resistance in melanoma. Cancer Discov.

[27] Liu F, Cao J, Wu J, Sullivan K, Shen J, Ryu B, Xu Z, Wei W, Cui R (2013). Stat3-targeted therapies overcome the acquired resistance to vemurafenib in melanomas. J Invest Dermatol.

[28] Hu W, Jin L, Jiang CC, Long GV, Scolyer RA, Wu Q, Zhang XD, Mei Y, Wu M (2013). AEBP1 upregulation confers acquired resistance to BRAF (V600E) inhibition in melanoma. Cell Death Dis.

[29] Gopal YN, Rizos H, Chen G, Deng W, Frederick DT, Cooper ZA, Scolyer RA, Pupo G, Komurov K, Sehgal V, Zhang J, Patel L, Pereira CG (2014). Inhibition of mTORC1/2 overcomes resistance to MAPK pathway inhibitors mediated by PGC1α and oxidative phosphorylation in melanoma. Cancer Res.

[30] Girotti MR, Pedersen M, Sanchez-Laorden B, Viros A, Turajlic S, Niculescu-Duvaz D, Zambon A, Sinclair J, Hayes A, Gore M, Lorigan P, Springer C, Larkin J (2013). Inhibiting EGF receptor or SRC family kinase signaling overcomes BRAF inhibitor resistance in melanoma. Cancer Discov.

[31] Holderfield M, Deuker MM, McCormick F, McMahon M (2014). Targeting RAF kinases for cancer therapy: BRAF-mutated melanoma and beyond. Nat Rev Cancer.

[32] Chapman PB, Hauschild A, Robert C, Haanen JB, Ascierto P, Larkin J, Dummer R, Garbe C, Testori A, Maio M, Hogg D, Lorigan P, Lebbe C, BRIM-3 Study Group (2011). Improved survival with vemurafenib in melanoma with BRAFV600E mutation. N Engl J Med.

[33] Colvin T, Gabai VL, Gong J, Calderwood SK, Li H, Gummuluru S, Matchuk ON, Smirnova SG, Orlova NV, Zamulaeva IA, Garcia-Marcos M, Li X, Young ZT (2014). Hsp70-Bag3 interactions regulate cancer-related signaling networks. Cancer Res.

[34] Doong H, Rizzo K, Fang S, Kulpa V, Weissman AM, Kohn EC (2003). CAIR-1/BAG-3 abrogates heat shock protein-70 chaperone complex-mediated protein degradation: accumulation of poly-ubiquitinated Hsp90 client proteins. J Biol Chem.

